# Correction: Understanding the formation mechanism of crystalline hydrated polymorphs of carbonic acid from CO_2_ clathrate hydrate

**DOI:** 10.1039/d6sc90010k

**Published:** 2026-01-12

**Authors:** Selene Berni, Demetrio Scelta, Sebastiano Romi, Samuele Fanetti, Frederico Alabarse, Bjorn Wehinger, Roberto Bini

**Affiliations:** a LENS, European Laboratory for Non-linear Spectroscopy Via N. Carrara 1 I-50019 Sesto Fiorentino Firenze Italy scelta@lens.unifi.it roberto.bini@unifi.it; b ICCOM-CNR, Institute of Chemistry of Organo Metallic Compounds, National Research Council of Italy Via Madonna del Piano 10 I-50019 Sesto Fiorentino Firenze Italy; c Dipartimento di Chimica “Ugo Schiff” dell'Università degli Studi di Firenze Via della Lastruccia 3 I-50019 Sesto Fiorentino Firenze Italy; d ELETTRA, Elettra Sincrotrone Trieste S.C.p.A, AREA Science Park Basovizza Trieste 34149 Italy; e ESRF, European Synchrotron Radiation Facility 71 Avenue des Martyrs, CS40220 38043 Grenoble Cedex 9 France; f INO-CNR, Istituto Nazionale di Ottica Via N. Carrara 1 I-50019 Sesto Fiorentino Firenze Italy

## Abstract

Correction for ‘Understanding the formation mechanism of crystalline hydrated polymorphs of carbonic acid from CO_2_ clathrate hydrate’ by Selene Berni *et al.*, *Chem. Sci.*, 2025, **16**, 22769–22780, https://doi.org/10.1039/D5SC02241J.

The authors regret that there is a typo in one of the temperatures given in the text of the article which may affect the reader's understanding. On page 22774, the sentence “At 278 K, the same temperature at which the lattice phonon modes appeared in the Raman spectrum […]” should actually read “At 287 K […]”. The Royal Society of Chemistry apologises for these errors and any consequent inconvenience to authors and readers.

